# Patient knowledge, experiences and preferences regarding retinoblastoma and research: A qualitative study

**DOI:** 10.1111/hex.13043

**Published:** 2020-02-29

**Authors:** Catherine Moses, Kaitlyn Flegg, Helen Dimaras

**Affiliations:** ^1^ Department of Ophthalmology & Vision Sciences The Hospital for Sick Children Toronto ON Canada; ^2^ The Department of Health Studies University of Toronto Toronto ON Canada; ^3^ Division of Clinical Public Health Dalla Lana School of Public Health University of Toronto Toronto ON Canada; ^4^ Department of Ophthalmology & Vision Sciences Faculty of Medicine University of Toronto Toronto ON Canada; ^5^ Child Health Evaluative Sciences Program SickKids Research Institute Toronto ON Canada; ^6^ Center for Global Child Health SickKids Research Institute Toronto ON Canada

**Keywords:** focus groups, patient and public involvement, patient engagement, patient‐oriented research, research engagement, research priorities, retinoblastoma, thematic analysis

## Abstract

**Background:**

We launched a patient engagement strategy to facilitate research involvement of the retinoblastoma (childhood eye cancer) community in Canada. To inform our strategy, we aimed to uncover the experiences with retinoblastoma, knowledge of retinoblastoma and research engagement among retinoblastoma survivors and parents.

**Methods:**

Focus groups were held in Toronto and Calgary, including both in‐person and remote participants (via videoconference). Discussions centred on experience with retinoblastoma, knowledge of the disease and engagement with research. Focus group transcripts were evaluated by inductive thematic analysis.

**Results:**

Four focus groups (3 in Toronto, 1 in Calgary) were held with a collective total of 34 participants. Retinoblastoma had a substantial impact on the life of participants, but overall, patients reported being able to adapt and persevere. Experiential knowledge of retinoblastoma was identified as distinct from the theoretical knowledge held by their clinicians. Participants indicated they often acted as a knowledge broker, communicating information about the cancer to their social networks. Participants were willing to engage in research as partners, but recognized barriers such as time and appropriate training.

**Conclusions:**

Patients view their experiential knowledge of retinoblastoma as valuable to improving care and directing research. There is a unique role for research engagement in meeting the educational needs of patients.

## 1 INTRODUCTION

Retinoblastoma is an aggressive childhood cancer of the eye.^1^, ^2^ Retinoblastoma is generally caused by a biallelic mutation of the *RB1* gene.^3^ Approximately half of retinoblastoma patients carry a mutation in the *RB1* gene in their constitutional cells. People with this heritable form of retinoblastoma have heightened risk of second malignancies later in life and a 50% chance of passing on the disease‐causing allele to their offspring.

Research on the health status of retinoblastoma survivors indicates that the disease has lifelong effects on daily life regardless of prognosis.^4^ The long‐term implications of a hereditary cancer syndrome, such as retinoblastoma, necessitate not only extended clinical follow‐up but also long‐term engagement with the health and research community. At the Hospital for Sick Children (Toronto), a study was conducted that examined knowledge, attitudes and experiences of Canadian retinoblastoma survivors and their parents, with regard to the genetics of the cancer.^5^ Unexpectedly, the study found that study participants were keenly interested in becoming more involved in retinoblastoma research.

Engagement of patients in other areas of health research has shown to increase democratization of the research process, study enrolment and retention rates,^6^ and broader community engagement.^7^ Results from such studies are more credible and acceptable to stakeholders,^6^, ^7^, ^8^ leading to improved clinical programmes.^9^ The term ‘patient’ refers to individuals with lived experience of disease and informal caregivers ^10^; for retinoblastoma, this could include parents, guardians, survivors, spouses, siblings and unaffected carriers.

Given the documented value of patient engagement strategies to bolster research,^6^, ^9^, ^11^, ^12^, ^13^, ^14^ we launched the Canadian Retinoblastoma Patient Engagement Strategy in 2016.^15^, ^16^ To inform the further development of this Strategy, we sought to examine the perspectives of retinoblastoma patients in Canada regarding their experiences, knowledge and preferences regarding retinoblastoma and research engagement. We rationalized that this information would help tailor strategy recruitment and engagement efforts, and develop novel research activities to meet the needs and preferences of the Canadian retinoblastoma patient community.

## 2 METHODS

### 2.1 Study question and design

This qualitative cross‐sectional study asked the question, ‘What are the experiences with retinoblastoma, knowledge of retinoblastoma, and research engagement among retinoblastoma patients?’ We employed the definition of ‘patient’ suggested by the Canadian Institutes for Health Research: individuals with personal experience of a health issue, including informal caregivers.^10^ Data were collected through focus group discussions. Research Ethics Board approval was received from the Hospital for Sick Children (REB# 1000054246).

### 2.2 Study setting

Focus groups were held in Toronto and Calgary, the rationale being that these two cities have the largest retinoblastoma communities. They took place in March and April 2017 at the Peter Gilgan Center for Research and Learning (Toronto) and the Marriott Downtown Hotel (Calgary, Canada).

### 2.3 Inclusion criteria

Participants were eligible to participate in the study if they were: (a) retinoblastoma survivors and/or the immediate family of someone diagnosed with retinoblastoma; (b) 18 years of age or older or considered an emancipated minor; and (c) residents of Canada.

### 2.4 Participant recruitment

Participants were recruited non‐randomly. Physicians, allied health‐care providers and advocacy groups were invited to disseminate a participant recruitment letter to their retinoblastoma networks. A participant recruitment poster was also disseminated through social media (Facebook, Twitter and Instagram) targeted to the Canadian retinoblastoma community.

Interested participants contacted the study team by email or telephone. A study team member confirmed eligibility criteria, noted availability and reviewed study objectives and consent form with the individuals. Participants were then asked to sign up for focus group time slots on a study‐specific REDCap ^17^ electronic data capture tool hosted on the Hospital for Sick Children server. A unique study ID code was issued for each participant, and protected identifiable information was documented in a code‐breaking log, which was stored separately from study data. Once dates for focus groups were set, an email was sent to all participants with a copy of the consent form for their review, location details for in‐person participants and login details for remote participants.

### 2.5 Sample size

Ethical approval was granted to conduct up to 4 focus groups in Toronto and 2 in Calgary, collectively including a maximum of 60 participants. This was based on the estimated patient population in each city, balanced against an educated guess of how many focus groups might be necessary to reach theoretical saturation. Focus groups were conducted until a saturation of themes was reached.

### 2.6 Focus group structure

Each focus group included 6‐10 participants. Written informed consent was obtained from each participant prior to the beginning of each focus group. Participants completed a brief demographic questionnaire. Focus groups were video‐ and audio‐recorded, and 1‐2 study team members were present as note takers (CM, HD).

The facilitator (KF) directed the discussion using a focus group guide consisting of 12 open‐ended semi‐structured interview questions loosely based on previous patient engagement studies on rare diseases ^18^, ^19^ (Supporting Information S2). The questions covered four main areas of interest: (a) patient and family experiences with retinoblastoma; (b) perceived knowledge and knowledge gaps about retinoblastoma; (c) research interests and experiences; and (d) communication preferences related to research opportunities and results.

### 2.7 Data processing and analysis

Demographic data were summarized using descriptive statistics. Recordings were transcribed verbatim from audio recordings and non‐verbal communications referenced from written notes and video recording. Participants were de‐identified using participant's study ID codes.

A codebook was developed iteratively by study team members (KF, CM, HD). Each researcher independently coded one transcript to test for consistency and compared across the others. Once consensus was reached, data were coded by one study team member (CM), using NVivo QSR 11. Inductive thematic analysis was employed to derive common themes, following the study framework. An audit trail using memos was used in the initial coding phase, and discussions were held with the rest of the team (HD, KF) to ensure that the findings logically flowed from the raw coded data. This study adheres to the Standards for Reporting Qualitative Research (SRQR) guidelines ^20^ (Supporting Information S2) and Guidance for Reporting Involvement of Patients and Public‐2 (GRIPP2)^21^ (Supporting Information S3).

### 2.8 Researcher characteristics and reflexivity

Study team members were researchers from non‐clinical backgrounds with roles of research coordinator (KF), undergraduate research student (CM) and academic scientist (HD). The researchers acknowledged that their involvement in launching the Canadian Retinoblastoma Patient Engagement Strategy may have impacted participant responses, or shaped how the results were interpreted.

## 3 RESULTS

### 3.1 Study participants

#### 3.1.1 Demographic characteristics

Fifty individuals expressed interest in participating in the study. Of these, 34 were eligible and available to attend the focus groups. Four focus groups were conducted: 3 in Toronto and 1 in Calgary. Most participants participated in person (20/34, 59%), were female (20/34, 59%), aged 25‐39 (18/34, 53%) and lived in a large urban centre (15.34, 44%) (Table 1).

**Table 1:** Participant demographics

	n	%
All participants	34	100
Sex		
Male	14	41
Female	20	59
Age		
20‐24	1	3
25‐39	18	53
40‐54	12	35
55+	3	9
Primary language		
English	33	97
French	0	0
Arabic	1	3
Population group		
Caucasian	28	82
Chinese	1	3
South Asian/Black/Latin American	1	3
South‐East Asian	1	3
South Asian	1	3
Korean	1	3
Arab	1	3
Religion		
Christian	18	53
Jewish	1	3
Muslim	2	6
Traditional (Aboriginal) spirituality	1	3
No religious affiliation	9	26
Other	3	9
Current residence		
Rural area	4	12
Small population centre	7	21
Medium population centre	9	26
Large urban population centre	14	41
Moved to receive treatment for retinoblastoma		
Yes	1	3
No	33	97
Education		
Some secondary education (high school)	2	6
Secondary school (high school) diploma or equivalent	1	3
Postsecondary certificate or diploma	7	21
Bachelor's degree	11	32
University certificate, diploma or degree above bachelor's degree	13	38
Employment		
Employed	20	59
Unemployed and looking for work	2	6
Unemployed and not looking for work	1	3
Homemaker	4	12
Employed/student	2	6
Employed/homemaker	1	3
Retired	2	6
Other	2	6
Marital status		
Married	23	68
Living common law	2	6
Widowed	1	3
Separated (but still legally married)	2	6
Divorced	1	3
Single (never legally married or marriage legally annulled)	5	15
Income		
<$29 999 CAD	1	3
$30 000‐$49 999 CAD	5	15
$50 000‐$79 999 CAD	4	12
$80 000‐$99 999 CAD	3	9
$100 000‐$149 999 CAD	8	24
Over $150 000 CAD	12	35
No response	1	3
Number of children		
0	6	18
1	10	29
2	6	18
3+	12	35

#### 3.1.2 Relationship with retinoblastoma

Ten participants identified as survivors (10/34, 24%). Twenty‐four were parents of an individual with retinoblastoma (24/34, 71%). One participant identified as both a survivor and a parent. One survivor was a grandparent of a child with retinoblastoma. One individual did not fall into a survivor or parent category, being an unaffected *RB1* gene mutation carrier, inherited from a survivor parent and passed on to an unaffected carrier child (1/34, 3%) (Table 2).

**Table 2:** Experience with retinoblastoma

	n	%
Relationship to retinoblastoma (RB)		
Survivor	10	29
Survivor (no children with RB)	8	24
Survivor + parent of child with RB^a^	1	3
Survivor + grandparent of child with RB	1	3
Parent	24	71
Unaffected parent of child with RB	23	68
Survivor + parent of child with RB^a^	1	3
Other	1	3
Unaffected carrier^b^	1	3
Survivors		
Time since diagnosis		
20‐29 y ago	3	30
30‐39 y ago	4	40
50‐59 y ago	1	10
No response	2	20
Laterality of retinoblastoma (Survivor)		
Unilateral	7	70
Bilateral	2	20
No response	1	10
Parents		
No. of children with retinoblastoma		
1	22	92
2	0	0
3+	2	8
Time since diagnosis		
<1 y ago	3	13
1‐5 y ago	9	38
6‐10 y ago	9	38
10+ y ago	3	13
Laterality of retinoblastoma		
Unilateral	8	33
Bilateral	15	63
Uncertain	1	4

^a^ This person counted in both survivor and parent group.

^b^ Child of RB survivor, and parent of unaffected carrier.

Time since diagnosis for survivors ranged between 20 and 59 years prior to the study date. Most survivors in the study had unilateral retinoblastoma (7/10, 70%) (Table 2). Of the parents, most had one affected child (21/24, 88%); most had bilateral retinoblastoma (15/24, 63%). Most children were diagnosed during the last decade (Table 2).

#### 3.1.3 Experience with research

Most participants (or their children) had prior experience with research (22/34, 65%) (Table 3). Their research role (or their child's) was mainly as a study participant. The majority indicated they were inclined (9/34, 26%) or strongly inclined (15/34, 44%) to participate as retinoblastoma advocates. Most participants were either unaware of (12/34, 35%) or felt there were too few opportunities (11/34, 32%) to participate in research (Table 3).

**Table 3:** Experience and interest in research

	n	%
Previous experience with research (n = 34)		
Previous research experience (self or child)	22	65
No previous research experience (self or child)	12	35
Number of prior research studies (Self)		
1	7	21
2	1	3
3+	3	9
No response	23	68
Role in prior research studies (Self) (n = 11)		
Participant	10	91
Study coordination	0	0
Other	1	9
Number of prior research studies (Child)		
1	3	9
2	2	6
3+	2	6
No response	27	79
Role in prior research studies (Child) (n = 7)		
Participant	7	100
Other	0	0
Interest in being a retinoblastoma advocate (n = 34)		
1—Not interested	2	6
2—Somewhat not interested	2	6
3—Neutral	5	15
4—Interested	9	26
5—Very interested	15	44
No response	1	3
Opportunities to participate in retinoblastoma research (n = 34)		
Usually unaware of any opportunities	12	35
Too few opportunities	11	32
Just enough opportunities	9	26
Too many opportunities	0	0
No response	2	6

### 3.2 Themes

Common themes were identified and related to three main categories: (a) experiences with retinoblastoma, (b) knowledge of retinoblastoma and (c) research engagement (Table 4).

**Table 4:** Summary of Themes

Category	Subcategory	Theme
Experience with retinoblastoma	Personal/Individual	Retinoblastoma has routine medical, visual and psychosocial consequences
		Patients adapt and persevere
	Medical care	Patients are satisfied with their primary retinoblastoma care
		There are areas of care which can be improved
	Community	The retinoblastoma community is a source of support
		Connecting to the retinoblastoma community can be challenging
Knowledge of retinoblastoma		Patients rate their personal knowledge of retinoblastoma as above average
		Patients recognize certain limitations to their knowledge of retinoblastoma
		Patients act as knowledge brokers
		There is a need for high‐quality trusted sources of retinoblastoma information
Research engagement		Patients are motivated to engage in research
		Past research engagement is limited to passive participation or advocacy
		There are significant barriers to participating in research

#### 3.2.1 Experiences with retinoblastoma

Experiences with retinoblastoma tended to centre on experiences (a) at the personal/individual level, (b) with medical care and (c) with the retinoblastoma community.

##### Experiences with retinoblastoma: personal/individual

###### Theme: Retinoblastoma has routine medical, visual and psychosocial consequences

Participants described the difficulties of routinely being affected by retinoblastoma, throughout their lives. Many participants cited medical or vision effects of retinoblastoma. These included the experience of a second primary malignancy, hearing loss as a result of chemotherapy treatment, vision loss or pain while reading. Several mentioned the ongoing effects of having had an enucleation: maintenance of the prosthetic eye, reconstructive surgeries, implant replacement or requiring protective eyewear.

I mean obviously, retinoblastoma germline has long‐term issues, effects from treatment, hearing aids. I think everyone has something or other that you know. (Participant B8)

My daughter is completely functionally blind. Uh, so, that's a huge adjustment, you know, uh, where things are in the house, where I put things down on the floor, um, anywhere she needs to go she's not, you know, she can't find her way and, all over by herself. (Participant C2)

Other routine ways retinoblastoma affected the participants were related to psychosocial effects. Insecurities surfaced about the aesthetic effects of enucleation or the irregular orbit caused by radiation therapy. Some individuals experienced bullying at school or felt that their reduced vision precluded them from participating in certain activities. Some survivors spoke about how they had lived with awkward situations throughout their lives and had learned how to handle them. Others felt that the physical appearance of artificial eyes impacted them in social settings:

And, it was a struggle, it's not fun being you know, the girl with the funny eye, and you know people are looking at ya‐ I really don't like that, and it took me a really long time to get over it, and uh, I'm over it. (Participant A8)

Individuals with the heritable form of retinoblastoma discussed the long‐term implication and worry associated with passing the mutation on to their children. Fear of a second cancer or cancer recurrence was also a common experience:

It's really the secondary cancers that provide me the most worry and cause me the least sleep…not really being sure, you know, when do I need to hit the panic button? And when is this just something that's a normal, everyday kid thing? (Participant D3)

Survivors commonly noted that their parents tended to exaggerate effects of retinoblastoma:

I think it's affected my parents more than it's actually affected me, like, I don't know, I lived, a reasonably good life. Like, I got married, I'm getting divorced, when I got divorced I started boxing, now my mom's flipping out “oh what happens if they hit you in the eye, you only have one eye”. So like, you're more worried about that than I am! (Participant C6)

In some focus groups, the interaction between strong, high‐functioning survivors with parents who expressed worry for their affected child appeared to alleviate some of that worry:

Parent: [Retinoblastoma] didn't get in the way right? (Participant C5)

Survivor: It didn't get in the way, it wasn't a hindrance. (Participant C6)

Parent: It's just amazing hearing that. (Participant C5)

###### Theme: Patients strive for normalcy

Paediatric cancers, especially those with lifelong implications like retinoblastoma, can hinder a child's ability to maintain normalcy. This was a concern expressed by some participants, especially parents:

I don't want my child to be known as, ‘the one who had cancer’…there's so much that's part of identity, I really push that to the background. (Participant B8)

I want her to carry on a normal life. I don't want her to be focusing on, ‘I have one eye, I have cancer, I had cancer, I don't know about my future’ I want her just to, you know, full fledge ahead. (Participant P2)

And yet, participants described feeling like they were normal in almost all aspects of life despite their retinoblastoma diagnosis:

Life yeah, life is complex so there's no such thing as normal, but I've lived with retinoblastoma, I've got one eye and I've lived a good life! I'm living a good life. (Participant C6)

In fact I think I've had a better life than most of my friends that have two eyes so, um, I don't think it's a hindrance at all, it's just, it's me, it's part of me! (Participant C6)

Participants, mostly survivors, suggested that retinoblastoma and its effects became part of their identity:

*So, I look at it as, I'm extra special because I had super rare cancer and I'm a survivor, so I'm extra, extra special. (Participant A8)* Patients appeared resilient in the wake of the retinoblastoma diagnosis:

I'm just the guy with one eye and my daughter had cancer…I'm just going to rock some sunscreen and hope for the best…I don't see myself as a victim…I can deal with it. (Participant B10)

Some discussions suggested that affected individuals may overcompensate for retinoblastoma by striving for success and high achievement:

I think about that a lot because I excel at sports, and I'm successful at my job and I'm a very busy, multitasking person, and I think all the time, that I have a really hard time slowing down and taking breaks, but I wonder if that's just because of who I am anyways? Or if, the artificial eye and the cancers wire us to be more as overachievers. (Participant A3)

##### Experiences with retinoblastoma: medical care

###### Theme: Patients are satisfied with their primary retinoblastoma care

Participants reported positive experiences with their primary retinoblastoma care (active treatment and immediate follow‐up) team.

As far as the clinical stuff goes at [name redacted], everything was…fantastic, it feels like a family there. (Participant C1)

I've been so fortunate, we've both been so fortunate, but, we've just had such great experiences with all of our doctors and nurses. (Participant C1)

Despite participants recognizing limitations in their retinoblastoma knowledge, they reported satisfaction with the information they received from their primary retinoblastoma care team.

Dr [name redacted] and her team did a great job explaining a lot of the medical things. It was amazing how overwhelming it was and yet we were able to get the information we needed. (Participant C5)

###### Theme: There are areas of care which can be improved

Despite largely positive experiences during primary care, participants suggested the need for enhancements in psychosocial support, ophthalmology follow‐up, oncology second cancer screening and transition to adult care, and communication between care providers.

Participants expressed that psychosocial support had been largely missing from their care. They suggested that such care should be provided by specialists within the retinoblastoma clinic, to avoid having to explain their medical history to a clinician outside the circle of care:

Then you go to the social worker and they say if you have insurance, then you can go get a psychologist. But you have a whole team of doctors here that understand what we've gone through. I'm more than happy to go on paying for private support, but…we're going to spend the first three appointments going through everything that we've gone through. (Participant B9)

Other comments were related to long‐term follow‐up. Some participants also emphasized the desire for an improved transition from paediatric to adult care. Some participants felt that there was a lack of follow‐up by the retinoblastoma medical community.

After I was no longer a patient with [the hospital] there was no follow up. It was only when I wanted to have a family, that I approached [the hospital] and asked questions. ‘What are the genetic risks? What are the options out there?’ (Participant A3)

Participants noted that they would like surveillance protocols to detect second cancers, noting that taking on that responsibility themselves was a source of stress.

Well you gotta think about the stress that causes the parent right? If I'm supposed to notice, I don't know what's happening in his bones and what's happening in his skin and it puts a lot of pressure on us. (Participant B11)

The issue of geographical location of residence as affecting quality of care was brought up by some. This was mainly a concern to those living outside Toronto.

I think the other thing is, and maybe I'm just disconnected, but Toronto's got a wealth of support for [retinoblastoma] families, the child specialists, the genetic counselling, all of that kind of stuff. I don't find that in Alberta…when you're not from there and you're living here…there are gaps. (Participant D9)

##### Experiences with retinoblastoma: community

###### Theme: The retinoblastoma community is a source of support

The overall feeling of participants is that others affected by retinoblastoma, or the ‘retinoblastoma community’ is a source of support.

So I've found comfort in those Facebook groups, I don't know if that's important to anyone else, maybe to pass on to their kids, I know there's an RB moms group and RB dads group and all that discussion that goes on online, and could be helpful to establish a bit of a community. (Participant A3)

###### Theme: Connecting to the retinoblastoma community can be challenging

With such a small community of retinoblastoma patients and caregivers, connecting to this community was challenging for some, particularly for families that live far from Toronto, the epicentre for retinoblastoma treatment in Canada.

So, we would go for treatment and then we would fly back home…we never really felt like we belonged in any sort of social support group, because we were just managing going back and forth. (Participant C3)

Some would have liked to be connected to other affected families through their caregivers and were disappointed that it could not be done (presumably due to confidentiality policies).

We've got the medical information, and Dr [name redacted] and her team did a great job explaining a lot of the medical things…but, the support side is lacking, we wanted to talk to someone else who's been in this situation…(but) there's no way that they could connect us to anyone. (Participant C4)

#### 3.2.2 Knowledge of retinoblastoma

##### Theme: Patients rate their personal knowledge of retinoblastoma as above average

Participants were asked to rate their knowledge of retinoblastoma on a scale from one (low) to ten (high) and explain their choices. Most perceived themselves as having higher than average knowledge, but rationalized that this was more knowledge than the average person, but still at ‘layperson’ level compared to clinicians. Several pointed to making an extra effort to educate themselves on the disease. A distinction was made between medical knowledge that clinical teams might have, compared to the experiential knowledge only those are affected by retinoblastoma can develop:

There's always going to be a gap between the people that went through it, and the people that work on it. We have the experience and understanding, you guys have the knowledge and technology and brains. (Participant A1)

##### Theme: Participants recognize certain limitations to their knowledge of retinoblastoma

All participants recognized they could learn more about retinoblastoma. Specifically, they identified areas where their knowledge was less than optimal: best practices for second cancer screening and prevention; psychosocial and other effects of current treatments; and new research directions for retinoblastoma.

There was clear distinction among participants: a group that wished to expand their knowledge of retinoblastoma and a group that did not. For those who wished to know more, they rationalized that they would be better prepared for decision making, reduce fear, and learn how to communicate more effectively with their families and other medical professionals.

We need the information so we can help to protect our kids, and advocate for our kids, because there are so many medical professionals who don't understand [retinoblastoma] and don't take those secondary cancer risks seriously. (Participant D3)

In contrast, those who did not want to receive more information rationalized that the extra knowledge might make them more fearful or anxious.

I think I know as much as I need to know. I don't like to dwell on it. It happened, it happened, and I hope it's something in the past. And I want to stay on top of it but I don't want to dwell on it cause, it's gonna eat up my life. (Participant B11)

##### Theme: Patients act as knowledge brokers

Several parents saw themselves as knowledge brokers, for example, to explain retinoblastoma and its consequences to family members or teachers.

I dread the beginning of every school year. You have to explain it, every time his eye falls out, you have to explain it to every new teacher. And you have to do it in such a way to make a big enough deal about it so that they, like, put sunscreen on him, and like, make a not big deal about it so they're not calling you for every single little thing. (Participant B9)

Participants also expressed a concern that they had to constantly act as their own advocates when interacting with health‐care providers outside of the retinoblastoma primary care team.

And it's also depending on which doctor you get, they know a lot about retinoblastoma, or they say they know a lot, they may not, um, they may just think it's childhood cancer…I've had to fight for secondary follow up all my life, um, it hasn't been easy. (Participant D4)

Some parents spoke about preparing their children to take on this responsibility for themselves in future:

What we always talk about with [my daughter] is that she's going to have to advocate for herself, and she's going to know more about this disease than the doctors will. (Participant D3)

##### Theme: There is a need for high‐quality trusted sources of retinoblastoma information

Participants spoke about the need for high quality of information about retinoblastoma from trusted sources. They outlined their struggles with accessing and understanding good quality information.

I feel like we get our information like fourth hand‐offs you know, on these Facebook groups and they say ‘well it was this study’ but we won't understand the study, and to read the study often you can't even get access to it, and even if you do get access to it I don't understand it. (Participant D6)

Some recognized that the online support groups could also be marred by incorrect information.

To anybody who experiences it now with Facebook, and support groups, I'm sure there can be a lot of contribution with incorrect information (Participant B8)

#### 3.2.3 Research engagement

##### Theme: Participants are motivated to engage in research

Participants indicated that they felt motivated and interested in being engaged with the retinoblastoma research community. Some wanted to help future generations or to express gratitude and respect to the retinoblastoma clinical team:

There were so many people that supported us when we were diagnosed, that to be able to give that back to new families, and to other families that are struggling is a huge motivator. (Participant D3)

The team saved my son's life… I'm forever in debt to [Hospital Q] and the team for saving my son's life and anything I can do to help out, I'd be more than happy just to help. (Participant C4)

Some recognized that by being part of the research teams, they could help influence research directions and be more likely to use results:

Parents' input is also invaluable to those doing the research, because I mean, this is our lives, so if we're able to voice our concerns and voice the areas that we find lacking…we can actually help, um, sort of, the use of the research, and apply it in to the care of our children, or ourselves… (Participant D8)

Others indicated that participation in research could enhance their personal knowledge and growth.

I think it's a win‐win for everybody right? I mean we, it's research, for you guys, but I never leave here without some new info you know? I'm always learning more and it's awesome for me right? I have questions to ask, and things to learn, and this is where it comes from. (Participant B11)

Some used the experience of their involvement in the current study to suggest psychosocial benefits to their participation:

This is the first time I've ever participated in a video conference like this or whatever, and um, I can now [say] that I'm really happy I did this. This is good. I think this is cathartic, and good to see success stories. (Participant C2)

##### Theme: Past research experience is limited to passive participation or advocacy

Participants described their prior participation in research studies, which was mainly centred on providing data or biological samples. Some struggled to recall the details of the studies:

We signed lots of paperwork at the time giving permission at the time for [my child] 's biological, um, samples to be used for research purposes…I'm not sure one hundred percent what all of them were. (Participant D3)

Other experiences with research involved using research results in their daily lives:

I've definitely made decisions based on, you know research and things about‐ that I've learned… (Participant D8)

##### Theme: There are significant barriers to participating in research

When asked about potential future research engagement, particularly as a member of a study team, some participants explained that their level of engagement was contingent on having necessary knowledge or skills, that lay language would facilitate their engagement and that access to findings would be helpful.

To do a proper research study, I would assume that you would have to have some background, and credentials to execute research. Right? (Participant B9)

English language would be good. ‘Cause half the time you can't understand what the heck that means! So, ‘dumb it down’ a bit. (Participant A8)

Some were also unsure of how they could be involved in research activities beyond simply advocacy:

I think I would (like to) be involved but I don't know how…Like the advocacy part of it‐ sure I'm gonna help inform people that it's possible right? Like I've seen pictures and we don't want to, but we tell people they should probably go and get things checked out. I guess I don't know how I could be involved in research? But I would. (Participant B10)

Some participants also explained that in the past, their involvement with the retinoblastoma community was limited by lack of information about events. They also shared that compensation was an incentive to be engaged with retinoblastoma research. Some participants explained that time constraints would restrict engagement with the retinoblastoma research community. Some spoke about geographical barriers to participating in research:

I used to participate in research a lot, but now that I'm kind of more isolated, um, any academic institutions, I feel like I could do more but, I've had to turn down some studies lately because I'm just not in Ontario. (Participant B3)

## 4 DISCUSSION AND CONCLUSION

### 4.1 Discussion

The purpose of this study was to uncover the patient experiences with and knowledge of retinoblastoma and associated research. The intention behind the study was to inform the development of the Canadian Retinoblastoma Patient Engagement Strategy.^15^, ^16^ The results reveal that the retinoblastoma patient community has a wealth of diverse experiential knowledge related to retinoblastoma from a variety of patient perspectives (eg heritable and non‐heritable, unilateral vs. bilateral, parent vs. survivor). Yet, regardless of the type of experience, the effects of retinoblastoma were uniform: all‐encompassing and lifelong. These experiences reflect important avenues on which future research could be based.

Participants' general satisfaction with their primary retinoblastoma care is likely reflective of the high cure rates in Canada.^2^ Yet, the participants pointed out an urgent need for health‐care teams to cater to their psychosocial care. This is consistent with other studies of paediatric rare diseases where parents report unmet social, informational, emotional and psychological needs.^22^ A prior study on retinoblastoma indicated that multidisciplinary teams help parents with emotional support and coping with treatment, as do peer support groups, during times of stress.^23^ Following the current study, we conducted a priority setting workshop with patients, researchers and clinicians, which revealed that improving psychosocial care for families affected by retinoblastoma is one of the top 3 Retinoblastoma Research Priorities in Canada (publication pending).

There was also a strong sentiment from participants on the need of long‐term follow‐up into adulthood, particularly for the detection and prevention of second cancers. Studies indicate that the long‐term follow‐up of paediatric cancer survivors in survivorship programmes has substantial benefits, including the prevention or reduction of long‐term cancer‐related effects, assistance in transitional care and improvements in holistic care that address psychosocial and practical medical issues.^24^, ^25^, ^26^ However, system‐based barriers to the implementation of such programmes include limited resources and low institutional commitment. Clearly, participants in this study felt more must be done to overcome the barriers which currently preclude consistent long‐term care for retinoblastoma survivors in Canada.

An interesting theme in the Calgary focus groups was that some participants felt too far removed from the perceived expert care available in Toronto. The disparities in cancer care access have been studied in Canada, indicating certain groups are at risk for inequitable access; geographically, this difference is usually between those living in rural vs. urban settings.^27^ The differences between urban centres, such as Calgary and Toronto, have yet to be studied. The Canadian Retinoblastoma Guidelines outline the recommended and mandatory services in retinoblastoma treatment centres ^28^; further study of retinoblastoma burden cross‐country may be necessary to improve capacity in urban centres outside of Toronto.

Parents and survivors indicated they often find themselves in the role of knowledge broker, to explain retinoblastoma to those outside the retinoblastoma community, such as extended family members or teachers. Participants recognized need for high‐quality and reliable information, but in an understandable format. In a prior study, online patient education materials in retinoblastoma were analysed for readability and found to be written at a higher grade level than recommended for patients, possibly interfering with their interpretation.^29^ Participants suggested that online materials were more prone to error, and preferred the information to come from a trusted source like their clinician. Consistent with this, a prior study revealed that patients rated online sources as least important for their learning and health‐care providers as most important.^30^ Interestingly, study participants noted that their knowledge could be enhanced through their participation on research teams. We infer then that patient partnership in research may have a role to play in improving knowledge of patients and families, creating another trusted source for information. The value of patients informing research has been demonstrated in the literature on patient engagement,^6^, ^9^, ^11^, ^12^, ^13^, ^14^ and this study reinforces that notion. Our participants expressed interest in joining research teams in future. Practically, they recognized barriers to their participation, such as time, training and compensation, consistent with what has been reported previously.^9^, ^31^ With the recent focus of the Canadian Institutes for Health Research on engaging patients in research, there are increased training opportunities for patients and scientists on how to form effective partnerships, and grants specifically focused on engaging patients in research. These findings have now been incorporated into the Canadian Retinoblastoma Patient Engagement Strategy.^32^ To address issues of training and compensation, a paid patient‐in‐research role was developed within the senior author's research team. The patient in research is fully embedded within the research structure, contributing to research design and implementation, and serves a direct link to the patient community. The Canadian Retinoblastoma Research Advisory Board meets annually to govern the strategy, with working groups led by patient and non‐patient pairs, who advance efforts to engage patients on projects that advance joint research priorities. Working group activities are sustained via monthly teleconferences, and modest stipends are provided to patient partners as a token of appreciation for their contributions. These activities are currently being evaluated using the Public and Patient Engagement Evaluation Tool.^33^

When parents are well informed, their children grow to be survivors who are well informed.^34^ Yet, a distinct portion of our participants indicated they did not wish to know more about retinoblastoma, perceiving it to only add to their anxiety and discomfort. Perhaps the desire for ‘normalcy’, expressed by some participants, is at odds with learning more about retinoblastoma, as it could be a reminder of having cancer, something perceived as ‘abnormal’. This dichotomy in the patient population—those who wish to know more, and those who do not—will be challenging to navigate for medical and research teams. Individualized approaches may be necessary to provide only as much information as desired by the patient, while ensuring that they have the necessary knowledge to achieve the best possible outcome.

One limitation to this study is that the geographical location of participants was limited to just four Canadian provinces, all English‐speaking. Recruitment aimed to reach patients and families nationwide, and while we did not achieve that, participants represented different types of retinoblastoma diagnoses and treatment experiences, and other demographic characteristics, including a higher participation of males than seen in other qualitative studies in our field. As the Canadian Retinoblastoma Patient Engagement Strategy grows and is strengthened, we expect to reach patients in all parts of the country and increase geographical representation in this respect.

### 4.2 CONCLUSION

In conclusion, retinoblastoma has a substantial impact on the life of those it affects. Patients view their experiential knowledge of retinoblastoma as valuable to improving care and directing research as it is distinct from the theoretical knowledge of the cancer held by clinicians. Patients recognized that they have some knowledge gaps on retinoblastoma, and interestingly were divided on whether or not they wished to learn more or remain in the dark. For those who do wish to learn more, this study finds that there could be a unique role for research engagement in meeting educational needs of patients, in addition to informing unique and patient‐centric directions for research.

## CONFLICT OF INTEREST

The authors declare that they have no competing interests.

## AUTHORS' CONTRIBUTIONS

KF and HD conceived and designed the study; CM, KF and HD acquired, analysed and interpreted the data; CM drafted the manuscript; CM, KF and HD critically revised the manuscript for important intellectual content and approved the final version of the manuscript.

## ETHICAL APPROVAL

Research Ethics Board approval was obtained from the Hospital for Sick Children (REB# 1000054246).

## Supporting information

SupinfoS2

SupinfoS3

## Data Availability

The data that support the findings of this study are available from the corresponding author upon reasonable request.
